# A novel and validated 3D-printed method for the consistent and reproducible dry transfer of microorganisms for the determination of antimicrobial surface efficacy

**DOI:** 10.1128/aem.00802-25

**Published:** 2025-07-23

**Authors:** Emma Chareyre, Alexander J. Cunliffe, Peter Askew, Gillian Iredale, Abby Marchant, Andrew P. Dean, James Redfern

**Affiliations:** 1 Department of Natural Sciences, Manchester Metropolitan University 5289, Manchester, United Kingdom; 2 Industrial Microbiological Services Ltd, Hartley Whitney, United Kingdom; INRS Armand-Frappier Sante Biotechnologie Research Centre, Laval, Quebec, Canada

**Keywords:** touch transfer, non-submerged inoculum, antibacterial materials, copper, MRSA

## Abstract

Transfer of microorganisms by touch is one way microorganisms can move within an environment. Antibacterial materials offer a potential solution, but currently, methods that use a dry inoculum, which are, therefore, realistic to many examples of touch transfer, are lacking. This study developed a novel, reproducible method for dry transfer of bacteria, using novel 3D-printed apparatuses, and applied the method to antibacterial copper surfaces. Method validation was carried out by transferring MRSA to stainless steel coupons under varying parameters and conditions (bacterial concentration, donor surface water content, bacterial inoculum drying time on donor surface). Additionally, the method was used to quantify the bacterial transfer to copper coupons under varying relative humidity environments, while comparing to the widely adopted standard ISO22196. The method demonstrated reproducible transfer of MRSA to both stainless steel and copper coupons. Antibacterial efficacy of copper following dry deposition was reduced in drier relative humidity environments, with the greatest population reduction after 2 h observed at higher relative humidity environments (>60%, 2.32-log), compared to the mid (40%–60%, 1.29-log) and low (<30%, 0.66-log) relative humidity environments. In contrast, copper demonstrated significant antibacterial activity under ISO 22196 methodology (i.e., wet inoculum and humid). These results provide a validated, novel protocol for dry transfer of bacteria for antibacterial efficacy assessment and highlight that copper-containing surfaces require greater environmental moisture for enhanced antibacterial effects. This study demonstrates the need for new standards more analogous to the end-use of an antibacterial material.

**IMPORTANCE:**

The transmission of microorganisms between surfaces by touch contributes to an increased healthcare burden due to secondary infection and mortality rates. Antibacterial materials can help to reduce the transmission of bacteria between surfaces and form part of a wider infection control system. However, testing the efficacy of antibacterial materials often uses unrealistic conditions (e.g., using large volumes of liquid) that may provide data overemphasizing their antibacterial action when in use. Additionally, there is no current standard method for assessing antibacterial surfaces contaminated via touch. This paper describes and validates a novel method to reproducibly transfer microorganisms to a surface enabling realistic deposition. Furthermore, the validated method was applied to antibacterial copper surfaces that are capable of passing current standards due to the availability of liquid (that copper surfaces require to be antibacterial) and found a reduced antibacterial effect under more realistic conditions.

## INTRODUCTION

The transmission of microorganisms via dry touch transfer represents a key pathway to the spread of disease across the globe (1), particularly in the built environment, where the high frequency and movement of people accelerate these processes (2). A wide range of different surfaces and environments are potential donors of microorganisms deposited by dry touch transfer (3). Areas of particular concern are those with a high volume of people, such as residential and commercial buildings (4, 5), healthcare and industry (6, 7), and public transport (8). Within household environments, transfer of infectious disease occurs at a rate of between 6% and 60% (9) attributed to both person-to-person transmission (e.g., via coughing/sneezing) and surface-mediated transmission. Public transport also presents a high risk of transfer, with bus/train call buttons, handrails, and seating as notable frequently touched surfaces (10). Furthermore, touch transfer of microorganisms often occurs through food cross-contamination, in both domestic and professional kitchens (11, 12), which can lead to foodborne illnesses (13).

In healthcare environments, fomite-mediated transmission is involved in the increased prevalence of healthcare-associated infections (14), which are directly responsible for increased patient morbidity and mortality (15). Fomites are surfaces in hospitals and other care facilities that can become contaminated and act as reservoirs of microorganisms, contributing to the indirect transfer of pathogens between people, and the subsequent health and economic impacts that can result from this (16). For example, methicillin-resistant *Staphylococcus aureus* (MRSA) is a leading causative agent for surgical site infections in healthcare environments (17) and has been shown to transfer via fomites and healthcare workers (18).

There have been several research articles published on methods to simulate transfer of microorganisms by touch. Many of those articles looked at transfer rates of microorganisms (19–21), as well as factors affecting transfer (22). The dryness of the inoculum, the type of surfaces involved in the exchange, and the type of touch (time and pressure) in both directions (microbial donor surface to intermediary surface/intermediary surface to recipient surface) were highlighted as key elements (22). For example, the transfer rate of two human norovirus genotypes as well as murine norovirus, from gloved fingertips or stainless steel to gloves, stainless steel, or small fruits in both wet and dry conditions, was assessed, whereby dry transfer was lower than that of wet transfer (23). A common trait of the methodologies described involved applying a light pressure (e.g., 50 ± 5 g) applied by a gloved human finger on a weigh scale to perform the transfer of microorganisms between surfaces (20, 21, 23), increasing the method’s variability between laboratories and individuals as such a low weight may be difficult to apply without variation for extended periods of time. However, despite these limitations, these research articles provide a foundation of knowledge to develop a more standardized and reproducible method for touch transfer simulation.

Antibacterial materials (ABM) are being increasingly integrated into areas of high importance (24) to mitigate the survival and growth of microorganisms after transmission via touch transfer. Within hospital environments, ABM have been proposed as a method of reducing the spread of hospital-acquired infections via frequently touched surfaces, with bed rails, bed surfaces, and supply carts in an intensive care unit described as the most frequently touched (25). Additionally, as this is often a result of staff interaction, the transfer of microorganisms via gloved hands is a key consideration, with nitrile being among the most abundant materials for hospital gloves globally (26). A material can be considered antibacterial if it effectively contributes to log reductions of bacterial populations (27). Usually, 2–3 log-reductions (but up to 5 depending on the context) are necessary to claim a material as antibacterial (27). Many accepted standards exist to test a given material’s efficacy as an antibacterial, such as ISO 20743 for textile materials or ASTM E2149-13a for non-porous surfaces under dynamic conditions (28, 29). However, there are very few that test a material’s antimicrobial efficacy under non-submerged conditions, whereby there is no liquid present on the surface for the duration of the incubation period of the test. In some cases, for photocatalytic materials, a “wet” transfer (droplets of inoculum) may be applied to a surface but then allowed to dry rapidly (<15% relative humidity) for testing in non-submerged conditions (30). Alternatively, a material may be inoculated by dry transfer (such as hand contact) but then be placed in submerged conditions (e.g., to simulate a shower surface) (31). These variations may benefit the testing of specific end-use cases, such as submerged conditions for testing water piping or dry transfer for simulating patient hand contact on frequently touched surfaces in hospitals. As frequently touched surfaces in hospitals are generally considered dry environments and transfer occurs via touch, a method to simulate this end-use scenario would involve dry-transfer and a non-submerged environment.

The most widely used standard for efficacy testing of ABM is ISO 22196 (32) that was designed to assess the antibacterial efficacy of plastics and non-porous materials using wet transfer (and, thus, is not directly related to touch transfer efficacy analysis). While the standard provides high reproducibility (therefore, allowing comparison between materials), it has been highlighted within the international scientific community for not accurately representing what can be considered realistic environmental conditions of a hospital indoor environment. For example, ISO 22196 specifies 37°C and >90% relative humidity during the incubation period, while hospital environments are likely to be lower, e.g., 20°C and 40%–60% relative humidity (27). Additionally, as the method of depositing bacteria in standards such as ISO 22196 is via pipetting relatively large droplets (e.g., 400 mL), the methods do not replicate touch transfer, making it difficult to translate such efficacy data into supporting end-use application.

To combat these shortcomings, a recently published ISO standard, ISO 7581 (33), was designed to investigate antimicrobial materials under non-submerged conditions (although still wet transfer), by transferring small volumes of inoculum (1 µL) by pipette on to the test surface and spreading the droplet using the pipette tip. This is then left to fully dry, estimated to be between 3 and 10 min and incubated for 1 h and 24 h in moderate (35%–60%) relative humidity before recovery of the coupons. Although this standard does emulate environmental conditions more analogous to a wide range of end-use scenarios compared to ISO 22196, it still introduces variability through the spreading of the inoculum with the pipette tip, which may present difficulties when comparing results between laboratories (34). Furthermore, as the material is still inoculated via wet transfer, the standard is not representative of microbial touch transfer (that employs dry transfer and non-submerged conditions) and allows ABMs that are only active under moist conditions (e.g., copper) to exhibit an artificially inflated antibacterial effect (35).

The aim of this study was to design a novel method capable of reproducibly (defined as statistical similarity between 20 technical repeats performed over 2 biological repeats) performing dry touch transfer of bacteria on to surfaces and accurately assessing this transfer, as well as validating the novel method. To achieve the aim, this manuscript describes (i) designing and 3D printing a novel apparatus to maximize reproducibility of bacterial transfer to surfaces, (ii) validating the dry transfer of bacteria from a donor surface to a recipient surface via the novel apparatus over a range of variables (drying periods on donor surface or apparatus-attached glove sections, donor surface agar concentration, initial cell concentration on donor surface), (iii) using the validated method to assess the antibacterial efficacy of copper surfaces under conditions analogous to a hospital environment, and then (iv) compare these results to those generated using a currently available standard for non-porous surfaces (ISO 22196).

## MATERIALS AND METHODS

### Design of 3D printed stamp and stamp attachment table

To reproducibly simulate the dry touch transfer of microorganisms via gloved hand contact onto frequently touched hospital surfaces, a novel apparatus was required. To create this, design requirements of a stamping apparatus were:

- High stability to prevent movement during the stamping process, allowing even coverage of microorganisms across the surfaces,
- A flat stamping surface of a minimum 20 × 40 mm size for two coupons to be stamped simultaneously,
- The ability to apply varying weights to apply different pressures, therefore presenting the possibility to simulate a variety of end-use scenarios (e.g., lift call buttons, handrails, keyboards, etc.),
- The ability to attach sterile glove sections securely on to the stamp to simulate gloved hand contact.

**Fig 1:**
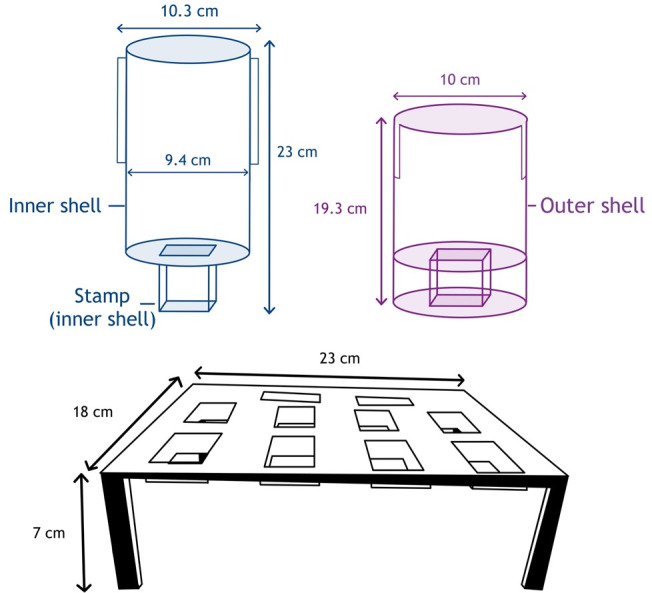
Schematic representation of the stamp and stamp attachment table (10 areas present to attach glove sections to).

The 3D-printed stamping apparatus (the stamp) was designed in two parts (inner shell and outer shell, Fig. 1). The inner shell includes the stamping surface and an internal area where weights can be placed. The inner shell is designed to be placed into the outer shell that provides stability against the surrounding surface (e.g., laboratory bench), with runners to ensure the correct orientation of the stamp as it is applied onto the surfaces.

To attach glove sections to the stamp in a sterile manner, a second apparatus (the stamp attachment table) was designed (Supplemental File A, Fig. A1E). The table was designed to have slots (with an underside ridge) to attach 65 × 50 mm glove sections to (using a small rubber band) where the stamp can move through and attach. The inner shell stamp was size 24 × 44 mm to ensure complete coverage of the stamping surface (20 × 20 mm metal coupons of either copper or stainless steel) and the slot on the outer shell was size 30 × 50 mm to allow the stamp to have slight freedom of movement (to avoid stamping the outer shell). Each slot (*n* = 10) of the stamp attachment table was size 26 × 46 mm to allow ease of retraction of the gloved stamp.

The stamp and table were designed in AutoCAD and exported to .stl file type (Supplemental File A, Fig. A1 A through D). This was then imported to BCN3D Cura (manufacturer-specific 3D-print software) for splicing (specifying each variable related to the 3D printer e.g. nozzle temperature), exported to .gcode, and transferred to the 3D printer via SD card.

Each section was printed using a BCN3D Sigma D25 3D-printer (BCN3D Technologies, Spain) according to the manufacturer’s instructions and polylactic acid (PLA, RS components, UK) was used as the printing material. Before each print, Magigoo adhesive (Farnell, UK) was applied to the print bed. Once printed, the necessary sections (Supplemental File A, Fig. A1 A through D) were constructed using non-corrosive silicone rubber (RS components, UK).

### Preparations required for transfer validation and antibacterial efficacy of copper testing

Tryptone Soya Agar (TSA, Oxoid, UK; 40 g/L deionized water) and Tryptone Soya Broth (TSB, Oxoid; 30 g/L deionized water) were prepared and autoclaved at 121°C for 20 min.

An overnight culture of methicillin-resistant *Staphylococcus aureus* NCTC 13143 (MRSA) was created by adding one colony from a TSA plate to 10 mL TSB and incubating for 24 h in an orbital incubator (Innova 2300, New Brunswick Scientific, United States) at 37°C shaking at 150 revolutions per minute, with a throw of 40 mm. The overnight culture was washed twice (with the following centrifugation settings: 2342 g for 10 min in a Sigma 3-16L centrifuge (Sigma-Aldrich, United States) with 0.15% Bovine Serum Albumin (BSA, Sigma-Aldrich; 1.5 g/L of deionized water). The overnight culture was then adjusted with 0.15% BSA to 0.500 (±0.01) OD at 600 nm (estimated to contain 1 × 10^8^ cells/mL) using a spectrophotometer (Jenway 6305 UV/Visible Spectrophotometer, Fischer Scientific) and calibrated using 0.15% BSA as a blank. The adjusted suspension was used as the working culture, unless 10^6^ cells/mL was specified (Table 1), in which case two serial dilutions of 1 : 9 were performed with 0.15% BSA.

**TABLE 1:** Factors tested for method validation^*a*^

Test ID	Drying time prior to touch (+: prior to transfer)	TSA concentration	Initial inoculum added to TSA
**1**	30 min drying time on agar	1×	10^8^ cells/mL
**2**	**90 min drying time on agar**	1×	10^8^ cells/mL
**3**	30 min drying time on agar	**1.5×**	10^8^ cells/mL
**4**	**30 min drying on agar + 5 min drying time on nitrile**	1.5×	10^8^ cells/mL
**5**	**30 min drying on agar + 1 min drying time on nitrile**	1.5×	10^8^ cells/mL
**6**	30 min drying time on agar	1.5×	**10^6^ cells/mL**

^*a*^ In bold are the primary changes of each test as compared to the first test.

**Fig 2:**
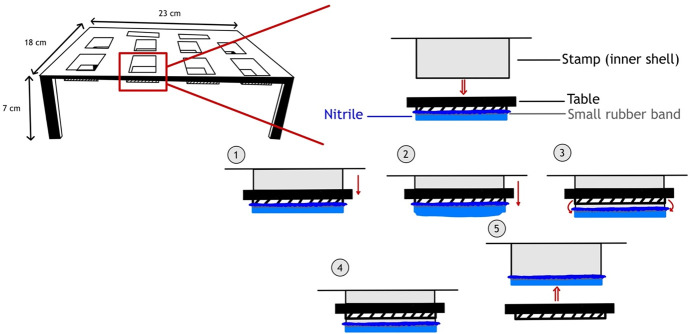
Schematic representation of the stamp attachment table when in use. 1. Stamp is pushed through the slot in the table. 2. Stamp pushes the nitrile section from the ridge of the table. 3. The small rubber band snaps in place around the stamp. 4. The nitrile section is secured around the stamp. 5. The stamp and nitrile section are removed from the slot in the stamp attachment table.

Nitrile glove sections of size 65 × 50 mm were cut from nitrile powder-free gloves (Fischer Scientific, UK), placed over the ridges on the underside of the stamp attachment table, and secured in place using a small rubber band (Primark, Ireland). The inverted stamp attachment table (gloved sections facing upwards) was then wrapped in a transparent polypropylene bag sealed with masking tape and irradiated with UV light for 30 min at 999,900 microjoules per cm^2^ using a CL-1000 Ultraviolet Crosslinker (UVP, USA) to sterilize the gloved sections. The stamp was attached to each glove section by passing the stamp through a slot of the stamp attachment table, allowing the rubber band to detach on to the stamp with the glove section, then removing together (Fig. 2). The nitrile glove sections were used to inoculate metal coupons. Once the 3D apparatus had been designed, the sterilization technique was validated by pressing the apparatus (with nitrile glove sections attached) directly on to TSA plates and incubating at 30°C for 48 h, whereby no growth was detected (images of the TSA plates after incubation are available in Supplemental File B).

Coupons were 20 × 20 mm 316 stainless steel to simulate a common inert hospital surface for transfer validation. For antibacterial efficacy of copper, stainless steel coupons were used to allow comparison of antibacterial effects (RS components, Corby, England) and EN1652 grade Cu-ETP copper as an antibacterial material (minimum 99.9% copper; RS components). Coupons were sterilized by wiping with 70% ethanol for 30 s and allowed to dry under sterile conditions for a minimum of 24 h (in sterile Petri dishes).

Coupons were recovered into the recovery medium of soya casein digest lecithin polysorbate (SCDLP) broth, made of 30 g/L TSB, 1 g/L L-α-lecithin (Acros Organics, Belgium), and 7.5 mL/L Polysorbate 80 (Acros Organics) in deionized water which acted as a neutralizer, validated to have no harmful effect to MRSA while being able to prevent any further antimicrobial efficacy of copper surfaces (method used and results showing validation of neutralizer available in Supplemental File C).

### Transfer validation to non-antibacterial surfaces

**Fig 3:**
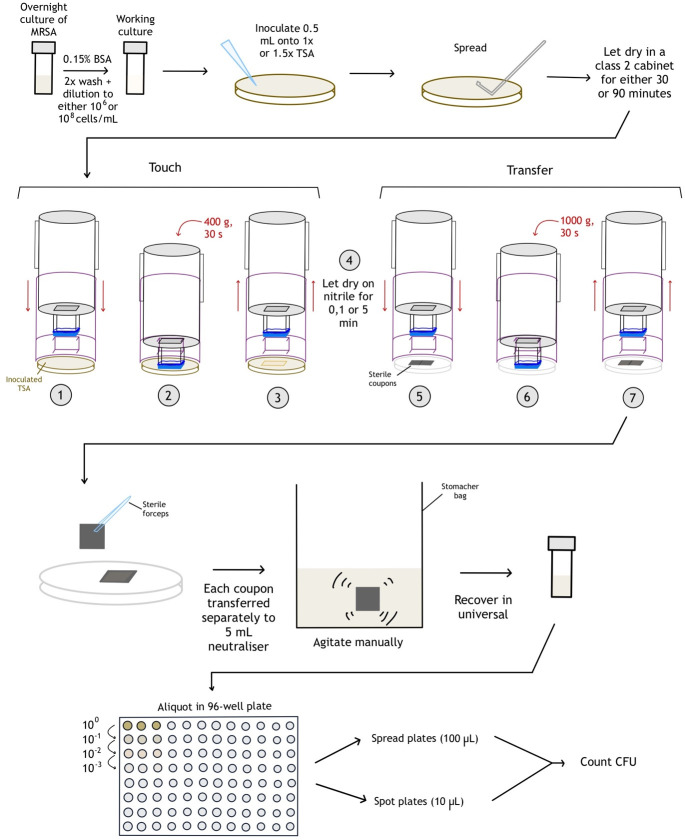
Protocol of the transfer validation. Prior to apparatus use, a TSA plate (1× or 1.5× concentration) was inoculated with a culture of MRSA at cell concentration 10^6^ or 10^8^ in 0.15% BSA, spread evenly, and allowed to dry for 30 or 90 min. The apparatus was used accordingly; Step 1: The inner shell is pushed through the outer shell; Step 2: The stamping surface touches the donor surface, and weight is applied on it; Step 3: The inner shell is removed from the outer shell; Step 4: The nitrile is allowed to dry under sterile conditions for a given duration; Steps 5–7: The same process as steps 1–3 is repeated on the recipient surface but with 1,000 g weight applied. Once complete, the coupons were then immediately recovered separately into 5 mL neutralizer and quantification occurred via dilution spread plate.

A visual protocol of the transfer validation procedure is shown in Fig. 3.

The stamp attachment table and 3D stamp were prepared as described previously. A TSA plate (either prepared to manufacturers specifications or 1.5× concentration to reduce water content of agar, Table 1) was inoculated with 500 µL of the MRSA working culture, spread on the plate with a sterile spreader, and allowed to dry for a set time prior to the initial transfer to the 3D printed apparatus (Table 1), with the lid removed in a class 2 cabinet.

The stamp device (inner shell post-glove attachment moving through the outer shell) was then placed on the inoculated agar plate and a mass of 400 g placed in the inner shell for 30 s, determined to be the maximum weight the TSA could support without splitting/breaking. Between 6 and 12 sterile stainless-steel coupons were placed into groups of two in sterile Petri dishes, with both coupons touching on one side. The stamp device and weight were removed and placed on to the coupons (two coupons per gloved stamp surface) with a mass of 1,000 g (chosen arbitrarily) in the inner shell for 30 s (Fig. 3). A video demonstration is available in Supplemental File D. In the case of additional drying time post-“touch”/pre-“transfer,” the stamp device was removed from the agar and placed for a set time (Table 1) inside of a sterile chamber (300 × 223 × 150 mm) at laboratory ambient temperature and relative humidity.

The stamped coupons were immediately recovered separately into 5 mL recovery media (SCDLP) in a sterile 80 mL stomacher bag (BA6040 standard bags, Seward, England), manually agitated for 30 s and the recovery media transferred into 30 mL sterile universal tubes (Fig. 3). The tubes were vortexed, and for each, 200 µL in triplicate was aliquoted using a pipette into a 96-well plate and then diluted 1:9, 1:99, and 1:999 in 0.15% BSA. For each triplicate aliquot (undiluted), round TSA plates were inoculated with 100 µL and spread with sterile spreaders. Each dilution was also spot plated (10 µL/spot) onto large (125 × 125 mm) square TSA plates and allowed to dry (Fig. 3). All plates were inverted and incubated at 37°C for 24 h, after which colony-forming units (CFU) were counted from the appropriate dilution, and final data were expressed as CFU/cm^2^.

Additionally, for test ID 3, the transfer rate was determined by quantifying both the bacteria on the nitrile (via recovering the nitrile sections directly into neutralizer) and that subsequently transferred to the stainless steel coupons. For this, all aspects of the test were the same as previously described except for the recovery of the nitrile.

### Use of the 3D printed stamp device to assess antibacterial efficacy of copper surfaces

**Fig 4:**
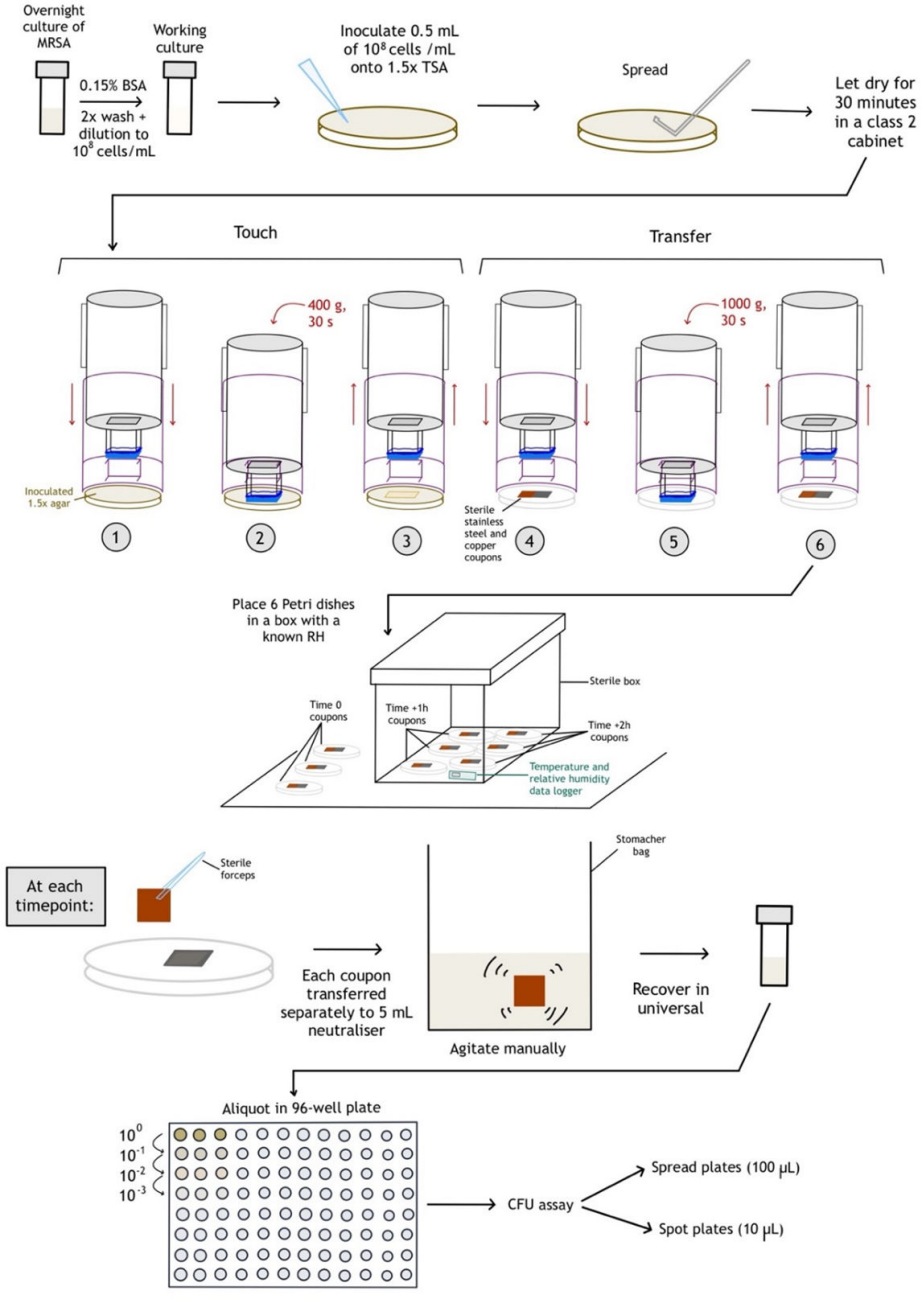
Protocol to assess antibacterial efficacy of copper using dry touch transfer. Prior to apparatus use, a TSA plate (1.5× concentration) was inoculated with a 10^8^ cells/mL culture of MRSA in 0.15% BSA, spread evenly, and allowed to dry for 30. The apparatus was used accordingly; Step 1: The inner shell is pushed through the outer shell; Step 2: The stamp device “touches” the donor surface and weight is applied; Step 3: The inner shell is removed from the outer shell; Steps 4–6: The same process as steps 1–3 is repeated on the recipient surface but with 1,000 g weight applied. Coupons were then transferred to a chamber with controlled environmental conditions (~20°C, <30/40–60/>70% relative humidity) and recovered at specific timepoints (0 h, 1 h, 2 h) into neutralizer separately, followed by quantification by dilution plate count.

A visual protocol for the antibacterial efficacy assessment of copper as an ABM following touch transfer using the novel method is shown in Fig. 4.

The stamp device, stamp attachment table, and coupons were prepared in the same manner as described previously, with the inoculum spread on 1.5× TSA the same as described in test ID 3 of the transfer validation (30 min drying time, 1.5× agar concentration, 10^8^ cells/mL). The agar plates were prepared as described above, moved to 4°C conditions until 24 h before use, and then returned to 20–24°C. The inoculated plates were placed in a laminar flow cabinet 30 min before use to dry.

Nine copper (antibacterial material) and nine stainless steel 316 (inert material) coupons were prepared and stamped as previously described (in each case, one copper and one stainless steel coupon were stamped simultaneously by placing next to one another), and three of each material were immediately recovered. To test the effect of relative humidity on post-transfer recovery, the remaining 12 coupons were transferred to a chamber (300 × 223 × 150 mm) that had been adjusted to either low (<30%), medium (40%–60%), or high (>60%) relative humidity by adding 200 g of saturated lithium chloride, potassium carbonate, or sodium chloride, respectively, using a porous polypropylene grid to place the Petri dishes containing the previously stamped coupons. A temperature and relative humidity data logger (RHT10; Extech, United States) was placed inside the chamber to record those parameters. Data were then saved as a .rec file and analyzed on the RHT10 software (Extech). Data relating to the temperature and relative humidity is available in Supplemental File E. At time points of 1 h and 2 h post-stamping, three of each coupon material were recovered for CFU analysis. To ensure that each coupon remained in the chamber for the intended duration (i.e., 0, 1, or 2 h), the process of stamping, placing the coupons in the chamber, and recovering the coupons occurred in sequence, with 3 min of separation between groups of coupons.

### Comparison to current standard: ISO 22196

In addition, ISO 22196 was also performed to compare the novel method to a currently available standard that utilizes a wet (droplet) deposition. The methodology used following ISO 22196 is as follows:

If used, all materials and solutions were prepared as stated above. Saline solution was prepared by adding 0.85% (wt/vol) sodium chloride to deionized water. Additionally, 1/500 nutrient broth was prepared by aliquoting 1 mL of nutrient broth (prepared as per the manufacturer’s instructions) to 499 mL deionized water. Both were sterilized by autoclaving at 121°C for 20 min. An inoculum of *Staphylococcus aureus* was prepared by transferring one colony from a TSA plate to perform a streak plate on another TSA plate and incubating at 37°C for 18–24 h. Colonies from this plate were transferred to 10 mL 1/500 nutrient broth to achieve an optical density of 0.5 ± 0.005 OD at 600 nm. This inoculum was then diluted 1 : 9 three times in 1/500 nutrient broth to create the working culture at a final cell concentration of ~2–7 × 10^5^ cells/mL. Six stainless steel and six copper samples were inoculated with 100 µL of the working culture and covered with a polypropylene square of size 18 × 18 mm sterilized in 70% ethanol for 30 s and allowed to dry overnight. Six coupons (three stainless steel, three copper) were immediately recovered into 5 mL neutralizer, manually agitated, the neutralizer transferred to 30 mL universal tubes, and vortexed to homogenize. The neutralizers were then diluted 1 : 9 in saline twice and 100 µL spread plated in triplicate on to TSA plates, incubated at 37°C for 24 h. The remaining three stainless steel and three copper coupons were transferred to a chamber (size 300 × 300 × 175 mm) set to >90% relative humidity (by adding deionized water, thus, that the bottom 5 mm of the chamber was submerged) and a grid added to allow samples within petri dishes to be placed on top while still allowing humidification of the air. After 24 h of incubation in the chamber, the recovery and plating process was repeated in the same manner as for the first six coupons.

### Statistical analysis and data visualization

Data were analyzed and visualized using R (version 4.4.0), on R-studio (version 2024.04.1 + 748). For graphical representation, R libraries “ggplot2” (version 3.5.1), “scales” (version 1.3.0), “Hmisc” (version 5.1-2), and “ggpubr” (version 0.6.0) were used. For all antibacterial efficacy data except ISO 22196, normality was verified using a Shapiro-Wilk test, and a Kruskal-Wallis test was run using the arithmetic means of technical repetitions. If the result was significant, pairwise Wilcoxon rank tests between conditions were completed to statistically compare the results. A *P*-value of *P* < 0.05 was considered significant. For ISO 22196 data, statistical significance was tested via a one-way ANOVA. Schematic representations of the 3D-apparatuses and visual protocols were hand-drawn on GoodNotes 5 (version 6.3.33).

## RESULTS

### Transfer validation to non-antibacterial surface

**Fig 5:**
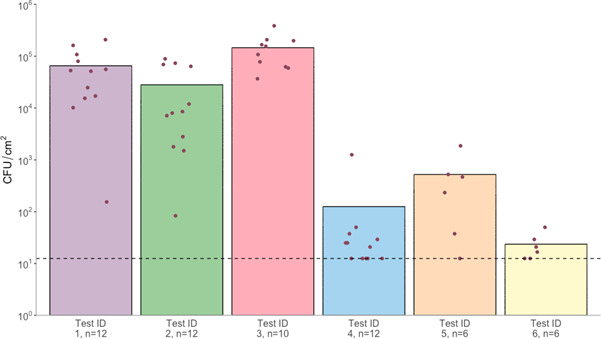
Recovery of MRSA from stainless steel coupons following transfer from apparatus attached nitrile glove-sections using each test ID (varying conditions for transfer). Each point represents one stainless steel coupon. Bars represent mean values. (•) represent arithmetic means of three technical repetitions. Dashed line represents limit of detection (1.25 × 10^1^ CFU/cm^2^).

A number of methods (described in Table 1) were tested to determine the best protocol to achieve high reproducibility and high overall transfer. The baseline test (Test ID 1) transferred an average of 6.55 × 10^4^ CFU/cm^2^ (Fig. 5), a transfer high enough that would allow for a log-scale comparison of antibacterial effects, but with a large variation between repeats (single coupon average range of 1.54 × 10^2^ to 2.09 × 10^5^ CFU/cm^2^). Test ID 2 (90 min drying time on agar prior to stamping) achieved similar results (Fig. 5), with an average of 2.81 × 10^4^ CFU/cm^2^ recovered from the coupons with a large distribution between repeats (range of 8.33 × 10^1^ to 8.92 × 10^4^). Test ID 3 (1.5 × agar concentration, 30 min drying time on agar, Fig. 5) presented an acceptable average recovered population (1.46 × 10^5^ CFU/cm^2^) as well as low variation between repeats (3.67 × 10^4^ to 3.86 × 10^5^). The use of additional drying times on the nitrile prior to transfer to the coupons (test ID 4/5) reduced the overall recovered populations (Fig. 5). A 5 min additional drying time (Test ID 4) recovered an average population of 1.19 × 10^2^ CFU/cm^2^, with a range of 1.25 × 10^1^ to 1.25 × 10^3^. The addition of a 1 min drying time (Test ID 5) resulted in an average of 5.21 × 10^2^ CFU/cm^2^ and a range of 1.25 × 10^1^ to 1.86 × 10^3^. Finally, test ID 6 (lower starting inoculum) as expected recovered the lowest populations of all tests with an average of 1.94 × 10^1^ CFU/cm^2^ and a range of 1.25 × 10^1^ to 5 × 10^1^ (Fig. 5). Information on individual coupon recovery can be found in Supplemental File F – Fig. F1. Overall, test ID 3 provided the highest recovery while also providing minimal variability between repeats and so was used as the method to assess the antibacterial efficacy of copper.

**Fig 6:**
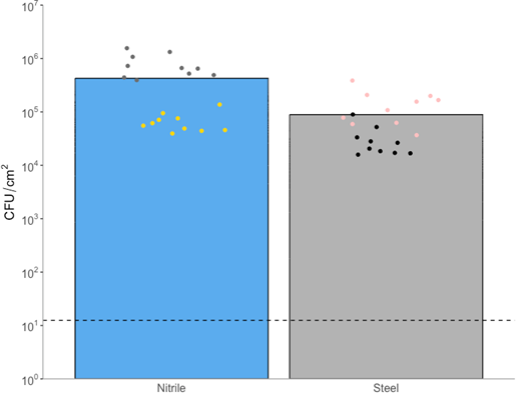
Recovery of MRSA from nitrile glove sections (pre-transfer to stainless steel) and stainless-steel coupons (post-transfer from nitrile glove sections) using the experimental set up described in test ID 3. Bars represent mean values. Each color represents a biological repetition, each of which represents the mean of three technical replicates. Dashed line represents limit of detection (1.25 × 10^1^ CFU/cm^2^).

To assess the transfer rate from the nitrile glove sections to the stainless-steel coupons, a further repeat of Test ID 3 was conducted and is presented alongside the previous data (Fig. 6, individual data available in Supplemental File F – Fig. F2). Populations that were recovered directly from the nitrile glove sections into neutralizer before transfer to the stainless-steel coupons were at an average of 6.67 × 10^5^ CFU/cm^2^ with a range of 1.37 × 10^5^ to 1.56 × 10^6^. Following transfer to stainless steel coupons, an average population recovery from the stainless-steel coupons of 8.89 × 10^4^ CFU/cm^2^, with a range of 1.58 × 10^5^ to 3.86 × 10^5^ was observed. This presents an overall transfer rate from nitrile to stainless steel of approximately 13.3% using this method.

### Antimicrobial efficacy of copper coupons

**Fig 7:**
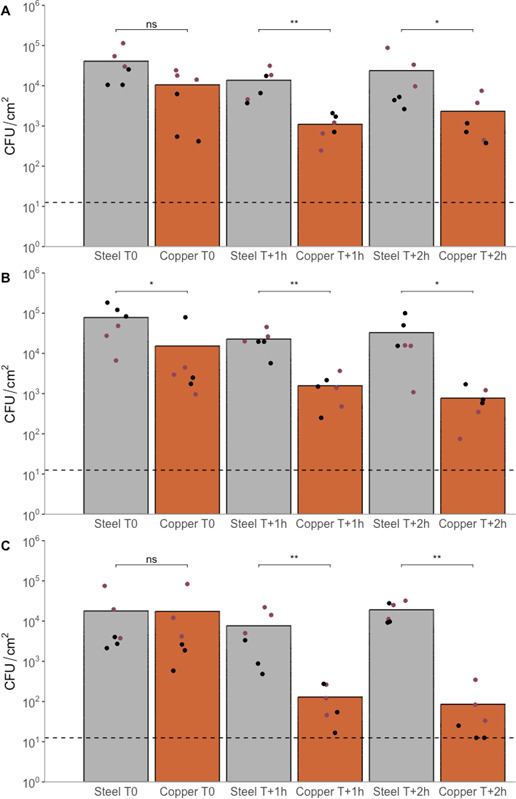
Transfer and survival of MRSA on stainless steel and copper coupons in (**A**) <30%, (**B**) 40%–60%, and (**C**) >60% relative humidity post-stamping. Bars represent mean values. (•) represent arithmetic means of three technical repetitions. Each color represents a biological repetition. A dashed line represents the limit of detection (1.25 × 10^1^ CFU/cm^2^). Statistical significance (Kruskal-Wallis test) was visualized as ns, “not significant,” “*,” *P* < 0.05; “**,” *P* < 0.01; “***,” *P* < 0.001; “****,” *P* < 0.0001.

The transfer and survival of MRSA to stainless steel and copper coupons is shown in Fig. 7. Overall, there is a similar initial transfer to both stainless steel and copper coupons although slightly lower recovery was observed from copper coupons at time 0 (and a significantly different population recovered from copper in one case in Fig. 7B, *P* = 0.0152).

Also, as surfaces containing copper are reliant on moisture to demonstrate antimicrobial action (as a medium for copper ion release and transport to cells to promote antibacterial efficacy), alongside assessing the desiccation tolerance of MRSA post-transfer, the survival and recovery of MRSA on stainless steel and copper coupons after incubation under varying relative humidity was performed (Fig. 7A: <30% RH/Fig. 7B: 40%–60% RH/Fig. 7C: >60% RH). A statistically similar recovery of the populations between stainless steel coupons at all time points (0 h, 1 h, 2 h) in all relative humidity conditions was observed, highlighting desiccation resistance of *S. aureus* on stainless steel and the non-antibacterial nature of stainless steel within this timeframe. For the lower RH, there was a 0.23-log reduction in population recovery after 2 h incubation (*P* = 0.1727), with a similar non-significant reduction observed at the mid RH (0.35 log, *P* = 0.2290) and increase at high RH (0.03 log, *P* = 1797). In contrast, varying population recovery was observed on copper coupons between the relative humidity conditions. As expected, the highest antimicrobial effect (and, therefore, reduction in bacterial populations recovered between time points 0 and T + 2 h) was observed under high relative humidity (higher moisture in the air) conditions (2.32 log, *P* = 0.005), while a statistically significant reduction in the populations recovered was observed at the mid relative humidity (1.29 log, *P* = 0.0087) and no statistical difference was observed in the low relative humidity (0.66 log reduction, *P* = 0.2403). This highlights the contribution of environmental moisture to the antimicrobial efficacy of copper-containing surfaces as well as the subtlety of antimicrobial efficacy changes under dry transfer and non-submerged conditions.

**Fig 8:**
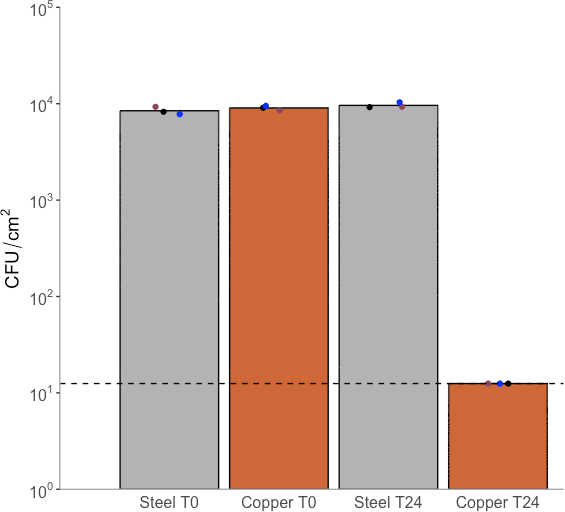
Survival of MRSA on stainless steel and copper surfaces when populations were recovered immediately and after 24 h when following ISO 22196 methodology. Different colors represent arithmetic means of three technical repetitions (repeats from a single coupon/neutralizer solution). Dashed line represents limit of detection (1.25 × 10^1^ CFU/cm^2^).

However, when assessing antimicrobial efficacy according to ISO 22196, a statistically significant reduction (*P* < 0.0001) in the populations recovered from copper surfaces to the limit of detection (LOD) was observed after 24 h (Fig. 8). Also, as expected, statistically similar populations were recovered from both stainless steel and copper coupons when recovered immediately (*P* = 0.5726) as well as those recovered from stainless steel immediately and after 24 h (*P* = 0.1181).

## DISCUSSION

The use of ABM can mitigate problems resulting from touch transfer of microorganisms. To determine the efficacy of ABM under conditions considered analogous to a hospital environment, methods for realistic dry transfer are needed. Therefore, the method described above has been developed and validated using a range of methodological variations demonstrating reproducible transfer of *S. aureus* to stainless steel and copper surfaces using the novel 3D printed stamping apparatus.

While other methods to simulate touch transfer to assess ABM appear in the literature (36, 37), these methods often involve human interaction that is difficult to standardize and, therefore, reproduce (e.g. pressure applied with a finger). While some methods may provide improved repeatability, access to identical equipment can result in inter-laboratory differences in results (38). Additionally, while research articles may describe the same aim (e.g., dry transfer of microorganisms), the methods employed can be different, reducing the comparability of the results. The touch transfer method described above can increase inter-laboratory reproducibility and accessibility of equipment by utilizing a bespoke 3D-printable apparatus. Furthermore, the apparatus allows high throughput of testing samples and can use different weights/pressures to simulate different touch transfer events while preventing contamination during the test procedure.

The initial validation of transfer of microorganisms to non-antibacterial stainless-steel surfaces (minimum of 1.00 × 10^4^ CFU/cm^2^ on average in test ID 3) highlighted that not only can bacteria be transferred between surfaces in a reproducible manner but also that an inoculum concentration realistic to a hospital contamination event can be transferred. For example, the typical environmental contamination in the indoor built environment on surfaces is between 10^2^–10^4^ cells in a given sampling area (usually 5–10 cm^2^) depending on the number of contamination events and cleaning protocols in use (39, 40). Furthermore, other methodological decisions were made to model intended end-use scenarios. When assessing the healthcare sector of the built environment, stainless steel is often used due to its inherent corrosion resistance, low-cost, high strength, and inert properties (41) and, therefore, is used as the “negative control” surface. Gloves (often nitrile) are often used within hospital environments to protect both the healthcare workers and patients from potential contamination events, and therefore, nitrile was used as a transferring/donor surface.

During development, different methodological choices resulted in high variability between repeats (test IDs 1 and 2) or a low transfer of bacteria onto the recipient surface (test IDs 4, 5, and 6). In the case of test ID 2, it may be that the bacteria did not transfer from the agar to the nitrile due to the longer drying period on agar, while test IDs 4 and 5 may not have transferred from the nitrile glove onto the stainless steel coupon due again to the period of time the inoculum was allowed to dry. This is further demonstrated in the transfer rate from nitrile to stainless steel, whereby the nitrile was recovered after stamping and was found to still harbor bacteria a level 3-logs higher than that recovered from the stainless-steel coupons.

Bacterial adhesion to surfaces throughout a range of end-use scenarios relating to the built environment is widely understood and involves stages of reversible attachment based on Van der Waals and hydrodynamic forces (42) that can strengthen over time (43), followed by transition to irreversible attachment as bacteria overcome the energy barrier to adhesion (44). This transition is dependent on the surface properties and may explain the disparities between test ID 2 and test IDs 4 and 5 due to the different properties of agar and nitrile surfaces. Specifically for nitrile, adhesion of bacteria has been demonstrated alongside potential surface modifications to decrease contamination (45). On the other hand, in test ID 1, incomplete drying of the inoculum on the agar surface caused some nitrile sections to appear visibly moist post-touch; this, therefore, does not provide conditions for bacterial adhesion and is not analogous to the conditions relating to that of non-submerged gloved hand contact and so cannot be considered for this method. However, it is possible that these conditions could be advantageous for simulating specific end use scenarios that have a higher level of moisture present (e.g., a public restroom where people often do not wash or dry hands completely [46]).

The higher concentration of agar within test ID 3 yielded consistent results that were reproducible, and above the minimal threshold for acceptance (1.00 × 10^4^ CFU/cm^2^ on average to allow for comparison of bacterial survival between surfaces), potentially due to the lower water content of the agar. These methodological conditions were investigated further, quantifying the CFU transferred onto both nitrile and on stainless steel in different instances, with the results also being satisfactory. Interestingly, although an inoculum size of 10^8^ cells/mL was applied to the agar surface, only ~10^6^ CFU/cm^2^ was recovered from the nitrile sections and ~10^4^ CFU/cm^2^ on the stainless-steel surfaces. The low efficiency of transfer between surfaces (20) may cause difficulties in using certain microorganisms using this method (e.g., yeast) where acquiring a high concentration inoculum may not be possible due to the larger size of individual cells and variations in cell density (47).

To assess the accuracy and reproducibility of the developed protocol (test ID 3), copper was used as a known and well-studied antimicrobial surface. Copper has been used throughout history as an antimicrobial, with evidence of its use in medical and food applications dating back to ancient Egypt (48). Copper has also been used within hospital environments to reduce bacterial load on frequently touched surfaces (49, 50). However, copper surfaces (among many other metal ion release-based materials) are highly reliant on moisture (liquid) in the surrounding environment to be able to exhibit their antimicrobial effect, as the metal ions require a medium to travel through to interact with the microorganisms (48). Therefore, a method that can reproduce dry touch transfer will better simulate the antimicrobial efficacy of copper in the built environment and, therefore, was selected for this study, using a range of different humidities (and, therefore, different amounts of moisture).

In addition to the differences in antimicrobial efficacy both between surfaces (e.g., stainless steel and copper) and between environments for the same surface, surface properties also impact the initial transfer of bacteria and, therefore, the spread of microorganisms throughout the built environment. In this study, there was a significant difference in the transfer at time zero between stainless steel and copper that may be explained due to the differences in overall structure of the metals. Surface micropattern was highlighted by reference 36 as a factor that may influence bacterial transfer rate and, therefore, could be an explanation for the observed transfer difference between the two metals. Recipient surface roughness was also highlighted as a factor that could impact microbial transfer although the focus was on the donor surface roughness rather than recipient (22). Despite both metals being non-porous, stainless steel has a smoother overall surface than copper due to grain size (as higher grain size is associated with higher surface roughness [51, 52]). The copper used had a grain size of 35 µm as per the manufacturer, whereas stainless steel has grain sizes between 10 and 25 µm (53). Therefore, the rougher surface of copper might have exhibited a minor impact on the transfer of MRSA and further emphasizes the importance of investigating methodological decisions based on individual materials that are being assessed.

When using this new method to assess antimicrobial efficacy of copper, a 1–2 log reduction in MRSA was found following 2 h of contact time, at low, medium, and high relative humidities. In contrast, results reported in the literature commonly show a greater antibacterial effect of copper, where complete kill of MRSA (from a 10^7^ cells/mL inoculum) can be observed between 45 and 100 minutes post-inoculation (54, 55). However, in such studies, the copper surfaces are inoculated with bacteria suspended in a droplet meaning the surface is wet. Other studies inoculate onto a surface and cover with a piece of polypropylene to increase the contact between the surface of the coupon and the inoculum, as described in Japanese standard JIS Z 2801 (56), which can also lead to a complete kill of *Staphylococcus aureus* with 99.90% copper (57). This is in agreement with the data generated in this study via ISO 22196 (a direct counterpart to JIS Z 2801), whereby completely kill is observed after 24 h due to the enhanced efficacy of the copper surfaces attributable to the availability of moisture. Few studies have assessed the antibacterial efficacy of copper via completely dry transfer and non-submerged conditions. In one case, a 4-log reduction in 120 s from a dry inoculum onto copper was observed due to membrane damage on *E. coli, B. cereus,* and *D. radiodurans* (58). To achieve a dry inoculum, the protocol specified streaking a coupon with an inoculated cotton swab and allowing to dry (reported under 5 s dry time). The authors concluded that the rapid decline in cell viability was due to irreparable membrane damage and rapid uptake of copper ions into the cells. A recent study also investigated dry deposition of *S. aureus* on copper and stainless steel using a nebulizer (59). The results were closer to those found in the present study, with a 1.03-log reduction 1 h post deposition. In another experiment, 1 µL of inoculum was spread onto coupons (considered “dry” 5 s post-inoculation) and observed a 5.76-log reduction within an hour (59). Given the contrasting results of the available literature, further study using reproducible techniques (e.g., inter-laboratory comparison trials) is required to determine whether there is a possibility of optimizing the methodology relating to applying a non-submerged inoculum to a surface. As much of the current literature assesses the antibacterial efficacy of copper with some moisture still present (even if described as a dry deposition), it is often not realistic to end-use scenarios where dry touch transfer is likely, which can be remedied by a dry transfer method such as the one described in this article.

For this study, MRSA was selected as it is often found in healthcare environments as well as other built environments and has been shown to survive on stainless steel for a minimum of 72 h (54, 60). Additionally, there are reports of MRSA being isolated in foods, mainly on products of animal origin such as meats and dairy (61, 62). Also, as MRSA is a gram-positive microorganism, it has high desiccation tolerance (63, 64) and is, thus, suited for study in non-submerged/dry conditions. However, further development of this method to include other microorganisms (e.g., gram-negative bacteria, yeast, viruses) or to other environments relating to MRSA would allow for more specific antimicrobial claims to be made based on the microorganism that is being targeted or environmental conditions of the environment.

Overall, this novel method offers a highly reproducible non-submerged transfer of bacteria, as opposed to other methods in the literature that either deposit the microorganisms in a droplet or via methods that allow for varying pressure to be applied to the surface (e.g., when using human fingers to perform transfer). The method presents a more realistic manner of assessing the bacterial transfer to surfaces via gloved hands and any potential benefits of implementing an antibacterial material. As this method offers high reproducibility, it can form the basis of a standardized testing method that evaluates materials in conditions analogous to those the material will experience when in use.

### Conclusion

The novel method developed in this work demonstrates reproducible transfer of bacteria onto a surface via a non-submerged inoculum. The novel 3D-printed apparatus designed, validated, and subsequently used in the method is easily accessible, and the recommended use limits human error. To demonstrate the use of the method, the antibacterial efficacy of copper following a dry/non-submerged inoculum in a range of relative humidity conditions was investigated. The work presented here provides a protocol that can be applied to other microorganisms to provide a reproducible method that would allow consistency of approach, facilitating cross-comparison of results at environmentally relevant conditions. In addition, this work facilitates the development of a novel standard to simulate touch transfer of microorganisms on to antibacterial materials that would allow for the simulation of a wide range of end-use scenarios (e.g., frequently touched surfaces in hospitals). Such a standard would allow for more accurate testing of such materials and better represent the efficacy a candidate material would exhibit once implemented.

### Article highlights

- There is currently no standard method to simulate touch transfer of microorganisms to antibacterial materials for efficacy assessment.
- A novel 3D printed apparatus was developed to reproducibly simulate bacterial touch transfer via gloved-hand contact.
- The novel method was validated by transferring (stamping) MRSA to stainless steel surfaces.
- The transfer rate between the nitrile glove sections (secured to apparatus) and stainless steel was calculated to determine optimal bacterial concentrations for the desired end-use scenario.
- Antibacterial efficacy of copper (as a currently approved antibacterial material) was evaluated in varying relative humidity post-stamping with apparatus.
- Copper has lower antibacterial efficacy in reduced relative humidity conditions.

## Data Availability

All 3D-print files are available for download at https://doi.org/10.5281/zenodo.15209857.

## SUPPLEMENTAL MATERIAL

Supplemental File A aem.00802-25-s0001.docx Images of 3D print files for stamping apparatus.

Supplemental File B aem.00802-25-s0002.docx Sterilization verification of stamping device via UV.

Supplemental File C aem.00802-25-s0003.docx Validation of neutralizer used for copper surfaces.

Supplemental File D aem.00802-25-s0004.mp4

Supplemental File E aem.00802-25-s0005.docx Temperature and relative humidity data when assessing antibacterial efficacy of copper surfaces.

Supplemental File F aem.00802-25-s0006.docx Individual recovery data (bacterial concentrations) from nitrile and stainless steel coupons.

Graphical abstract aem.00802-25-s0007.tif Visual diagram of the study.

ASM does not own the copyrights to Supplemental Material that may be linked to, or accessed through, an article. The authors have granted ASM a non-exclusive, world-wide license to publish the Supplemental Material files. Please contact the corresponding author directly for reuse.
