# Supplementary material for: A novel and validated 3D-printed method for the consistent and reproducible dry transfer of microorganisms for the determination of antimicrobial surface efficacy
Source: Appl Environ Microbiol. 2025 Jul 23;91(8):e00802-25. doi: 10.1128/aem.00802-25 (PMC12366365; doi:10.1128/aem.00802-25)
Supplement: Supplemental File A — Images of 3D print files for stamping apparatus. [file aem.00802-25-s0001.docx]

**Supplementary information A**


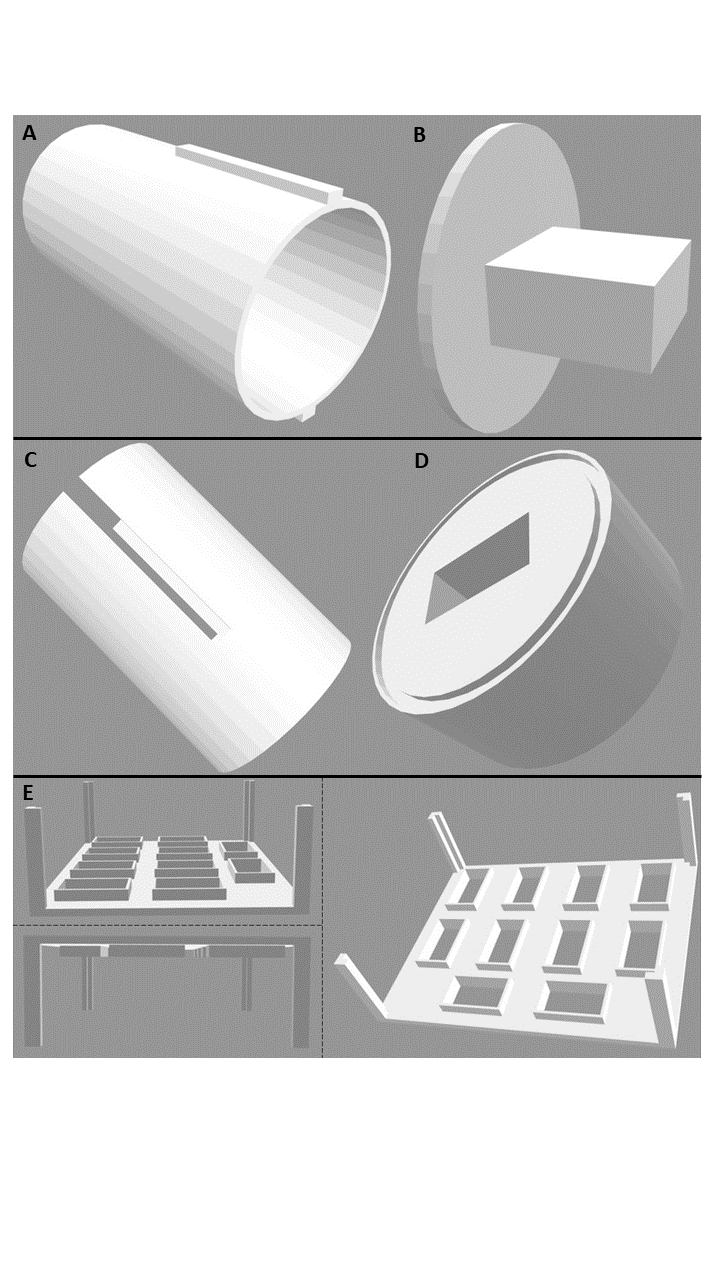


**Figure A1.** 3D-models (.stl file type) of the novel apparatuses after exporting from AutoCad. A-D: novel stamping apparatus, A-B: inner shell, C-D: outer shell, E: nitrile glove section attachment table
