# Supplementary material for: A novel and validated 3D-printed method for the consistent and reproducible dry transfer of microorganisms for the determination of antimicrobial surface efficacy
Source: Appl Environ Microbiol. 2025 Jul 23;91(8):e00802-25. doi: 10.1128/aem.00802-25 (PMC12366365; doi:10.1128/aem.00802-25)
Supplement: Supplemental File B — Sterilization verification of stamping device via UV. [file aem.00802-25-s0002.docx]

**Supplementary information B**

To ensure the sterility of the nitrile glove sections prior to transferring bacteria via touch transfer, the apparatuses were prepared as described in the manuscript and stamped directly on to a sterile tryptone soya agar plate with a weight of 400 grams for 30 seconds, repeated in triplicate (three separate nitrile glove sections each pressed on to one agar plate). The agar plates were then incubated at 30^o^C for 48 hours and assessed for colony formation. In all cases, no growth was observed.

**
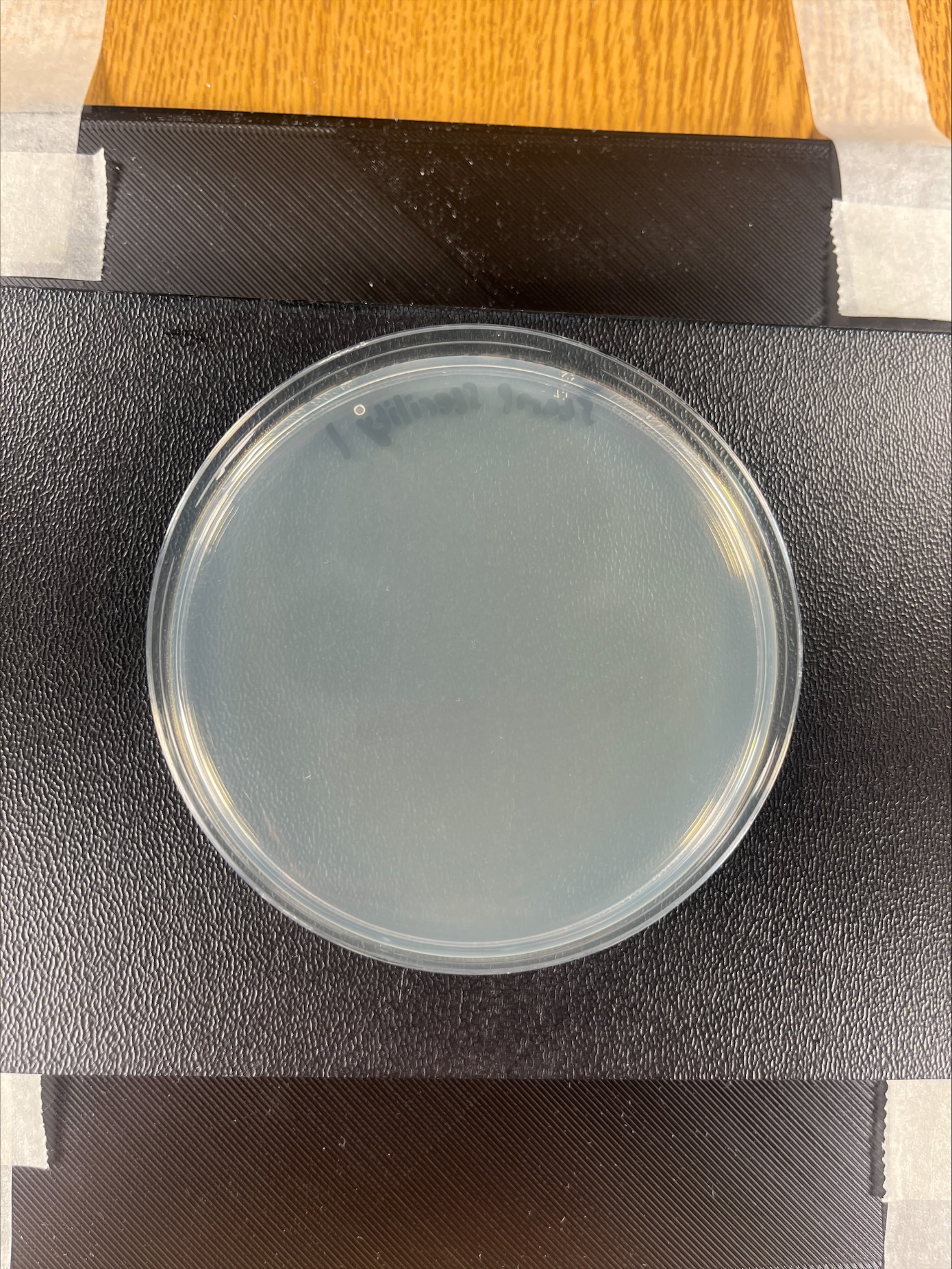
**

Figure 1 Tryptone soya agar plate pressed with UV sterilised stamping apparatus and incubated at 30^o^C for 48 hours, repeat 1.

**
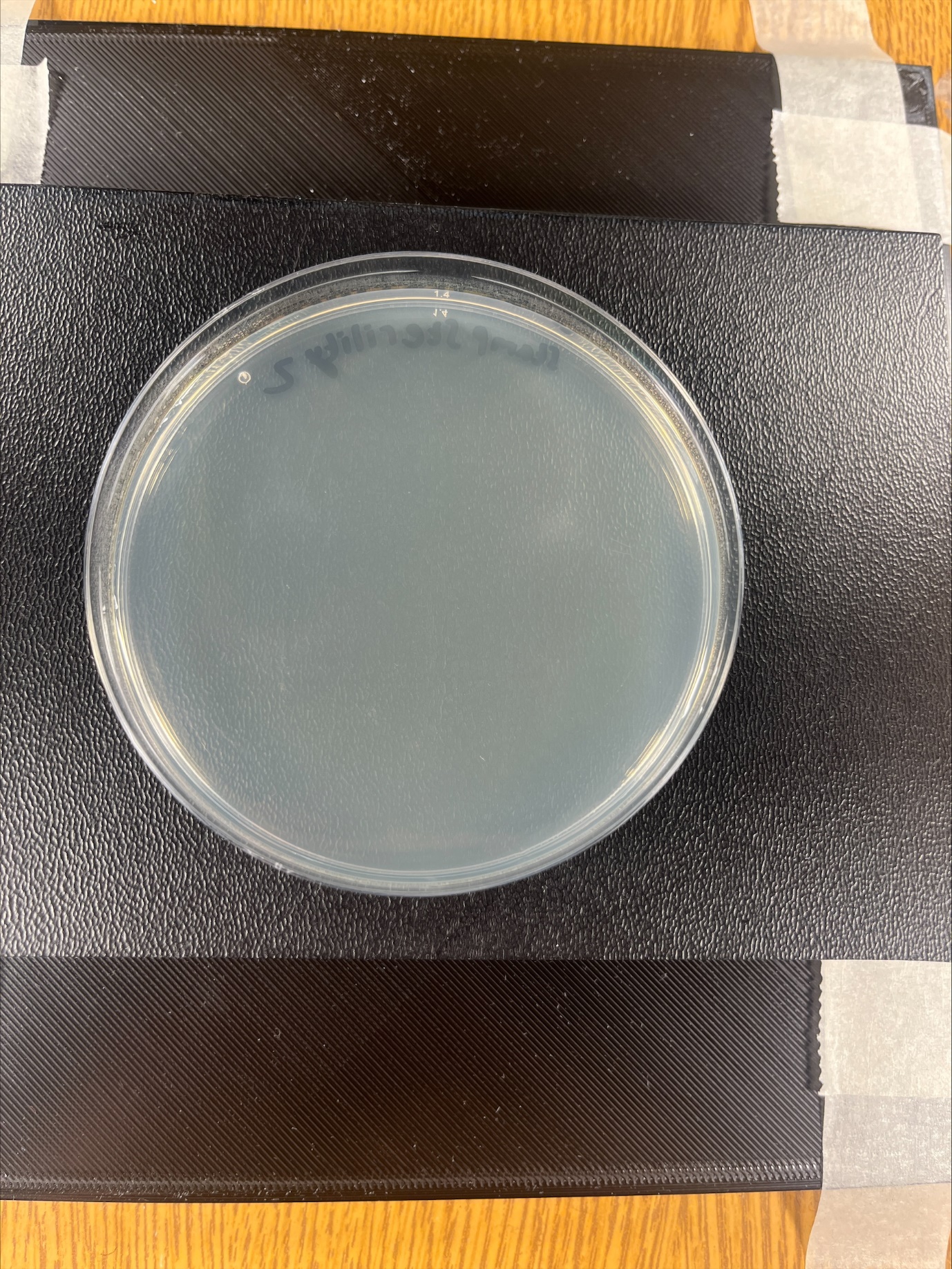
**

Figure 2 Tryptone soya agar plate pressed with UV sterilised stamping apparatus and incubated at 30^o^C for 48 hours, repeat 2.

**
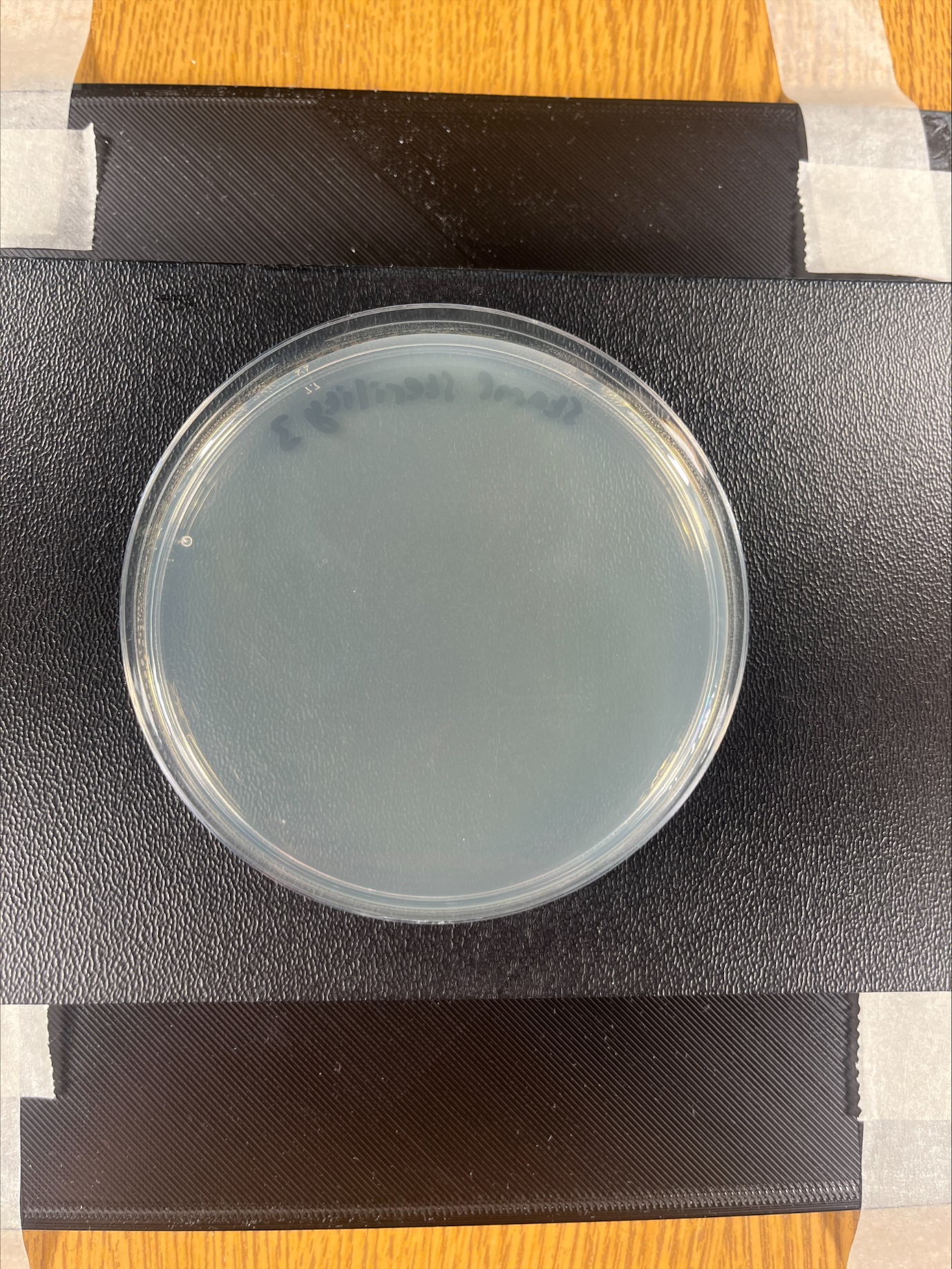
**

Figure 3 Tryptone soya agar plate pressed with UV sterilised stamping apparatus and incubated at 30^o^C for 48 hours, repeat 3.
