# Supplementary material for: A novel and validated 3D-printed method for the consistent and reproducible dry transfer of microorganisms for the determination of antimicrobial surface efficacy
Source: Appl Environ Microbiol. 2025 Jul 23;91(8):e00802-25. doi: 10.1128/aem.00802-25 (PMC12366365; doi:10.1128/aem.00802-25)
Supplement: Supplemental File C — Validation of neutralizer used for copper surfaces. [file aem.00802-25-s0003.docx]

**Supplementary information C**

**Neutraliser validation**

A validation test of the neutraliser (SCDLP) was performed to ensure no toxicity or continued antibacterial efficacy was occurring during the neutralisation period of the studies. The method was adapted from BS EN 1276:2019 (European Committee for Standardisation, 2019) for environmental condition requirement of the novel method.

**Method**

All materials and solutions were prepared as stated in the manuscript. The stamping device was prepared, and a sterile glove section used to stamp two copper coupons. Once stamped, the coupons were left in a sterile Petri dish for three hours within a chamber set to 20°C and 40 - 60 % relative humidity. Each coupon was transferred separately to an 80 mL stomacher bag (marked C), with 10 mL neutraliser added to each. Additionally, 10 mL sterile water (marked A) and 10 mL neutraliser (marked B) were transferred to 80 mL stomacher bags in duplicate. After 5 minutes, the coupons in stomacher bags C were massaged and a 100 µL inoculum of *Staphylococcus aureus* at ~1 × 10^4^ cells / mL added to each bag. The inoculum was diluted 1 : 9 to 10^-2^, 100 µL was then spread plated on to TSA in triplicate to confirm the initial cell concentration. Then, 30 minutes post-inoculation, the neutraliser from each bag was transferred to a 30 mL universal tube, homogenised with a vortex mixer, diluted 1 : 9 to 10^-1^ and 1 mL of each dilution and for each test added to a sterile empty Petri dish for pour plating with TSA.

**Results**

An acceptable pass rate of for both test comparisons (A-B / A-C) was achieved with 10^-1^ dilutions providing acceptable counts (30 – 300 colonies present) in all cases. Therefore, the neutraliser can be considered validated.

Table 1. Colony forming unit (CFU) counts recovered from (A) distilled water, (B) neutraliser, and (C) neutraliser with a copper coupon added, after inoculation with Staphylococcus aureus. Additionally, spread plating of the bacterial inoculum was performed to confirm initial cell concentration. All counts were performed from a 10^-1^ dilution of the respective samples (pour plate) and inoculum (spread plate). Averages of samples are displayed as per mL.

| Test | Repeat | Colony count | CFU / mL |
| --- | --- | --- | --- |
| Inoculated water (A) | 1 | 46 | 460 |
| Inoculated water (A) | 2 | 56 | 560 |
| Inoculated neutraliser (B) | 1 | 57 | 570 |
| Inoculated neutraliser (B) | 2 | 52 | 520 |
| Inoculated neutraliser with coupon (C) | 1 | 57 | 570 |
| Inoculated neutraliser with coupon (C) | 2 | 48 | 480 |
|  |  |  |  |
| Inoculum spread plates | 1 | 248 | 24800 |
| Inoculum spread plates | 2 | 237 | 23700 |
| Inoculum spread plates | 3 | 271 | 27100 |

| A average | B average | C average |  | A-B recovery rate | 106.9% |
| --- | --- | --- | --- | --- | --- |
| 510 | 545 | 525 |  | **A-C recovery rate** | 102.9% |

**References**

European Committee for Standardisation (2019) ‘EN 1276:2019 - Quantitative suspension test for the evaluation of bactericidal activity of chemical disinfectants and antiseptics used in food, industrial, domestic, and institutional areas.’
