# Supplementary material for: A novel and validated 3D-printed method for the consistent and reproducible dry transfer of microorganisms for the determination of antimicrobial surface efficacy
Source: Appl Environ Microbiol. 2025 Jul 23;91(8):e00802-25. doi: 10.1128/aem.00802-25 (PMC12366365; doi:10.1128/aem.00802-25)
Supplement: Supplemental File E — Temperature and relative humidity data when assessing antibacterial efficacy of copper surfaces. [file aem.00802-25-s0005.docx]

**Supplementary Information E**

To ensure the temperature and relative humidity within the chamber stayed within the desired range for each test, an RHT10 temperature and relative humidity datalogger was placed in the chamber for the duration of the test.

Below 30 % relative humidity

Both temperature and relative humidity (Figure 7) remained consistent throughout the test, with an average temperature of 23.70 °C (min – 22.60 °C, max – 24.30 °C) and relative humidity of 18.70 % (min – 14.40 %, max – 26.70 %).


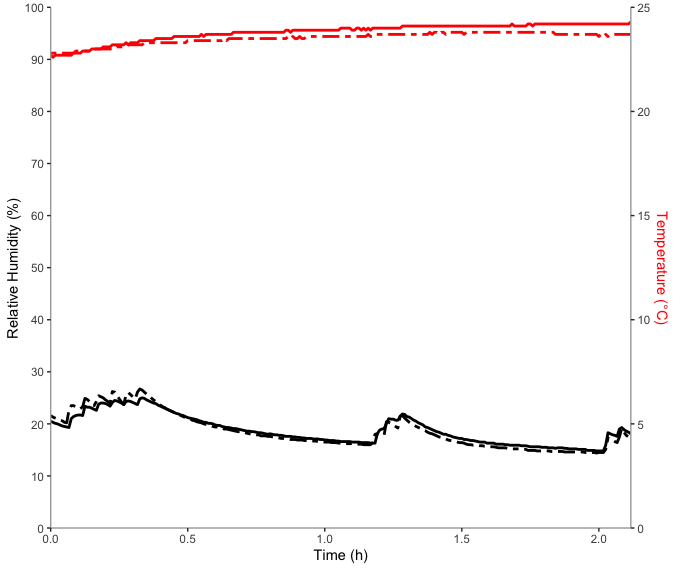


**Figure E1.** Temperature and relative humidity log of the chamber adjusted to < 30 % relative humidity to assess the antibacterial efficacy of copper coupons. Full lines and dashed lines represent two independent experiments.

40 – 60 % relative humidity

Both temperature and relative humidity (Figure 9) remained relatively stable throughout the test, with an average temperature of 22.50 °C (min – 21.90 °C, max – 23.00 °C) and relative humidity of 45.60 % (min – 44.40 %, max – 48.30 %).


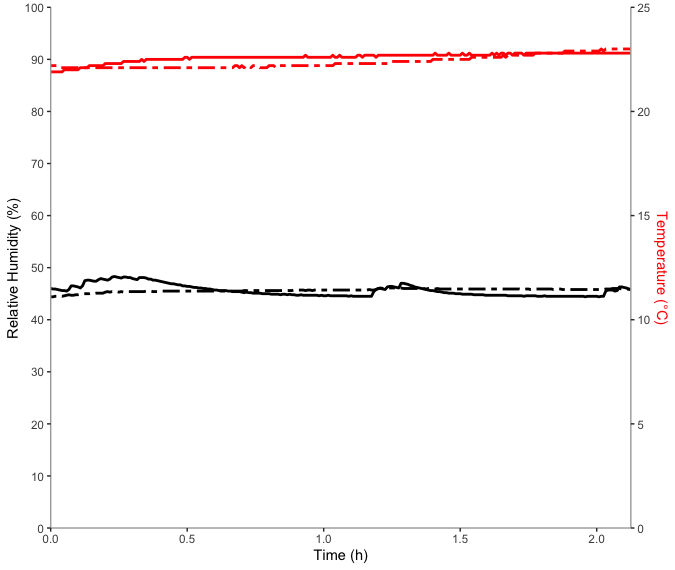


**Figure E2.**  Temperature and relative humidity log of the chamber adjusted to 40 – 60 % relative humidity to assess the antibacterial efficacy of copper coupons. Full lines and dashed lines represent two independent experiments.

Above 60 % relative humidity

Both temperature and relative humidity (Figure 11) remained consistent throughout the test, with an average temperature of 23.40 °C (min – 22.40 °C, max – 24.60 °C) and relative humidity of 69.60 % (min – 60.10 %, max – 71.80 %).


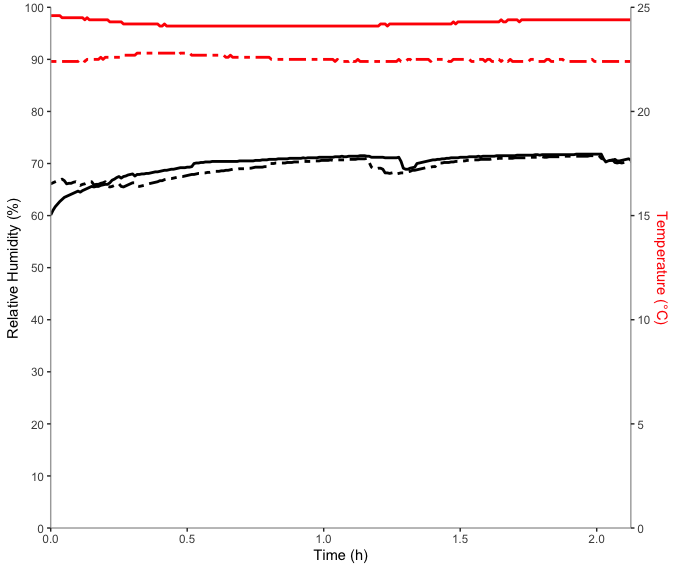


**Figure E3.**  Temperature and relative humidity log of the chamber adjusted to > 60 % relative humidity to assess the antibacterial efficacy of copper coupons. Full lines and dashed lines represent two independent experiments.
