# Supplementary material for: A novel and validated 3D-printed method for the consistent and reproducible dry transfer of microorganisms for the determination of antimicrobial surface efficacy
Source: Appl Environ Microbiol. 2025 Jul 23;91(8):e00802-25. doi: 10.1128/aem.00802-25 (PMC12366365; doi:10.1128/aem.00802-25)
Supplement: Supplemental File F — Individual recovery data (bacterial concentrations) from nitrile and stainless steel coupons. [file aem.00802-25-s0006.docx]

**Supplementary information F**


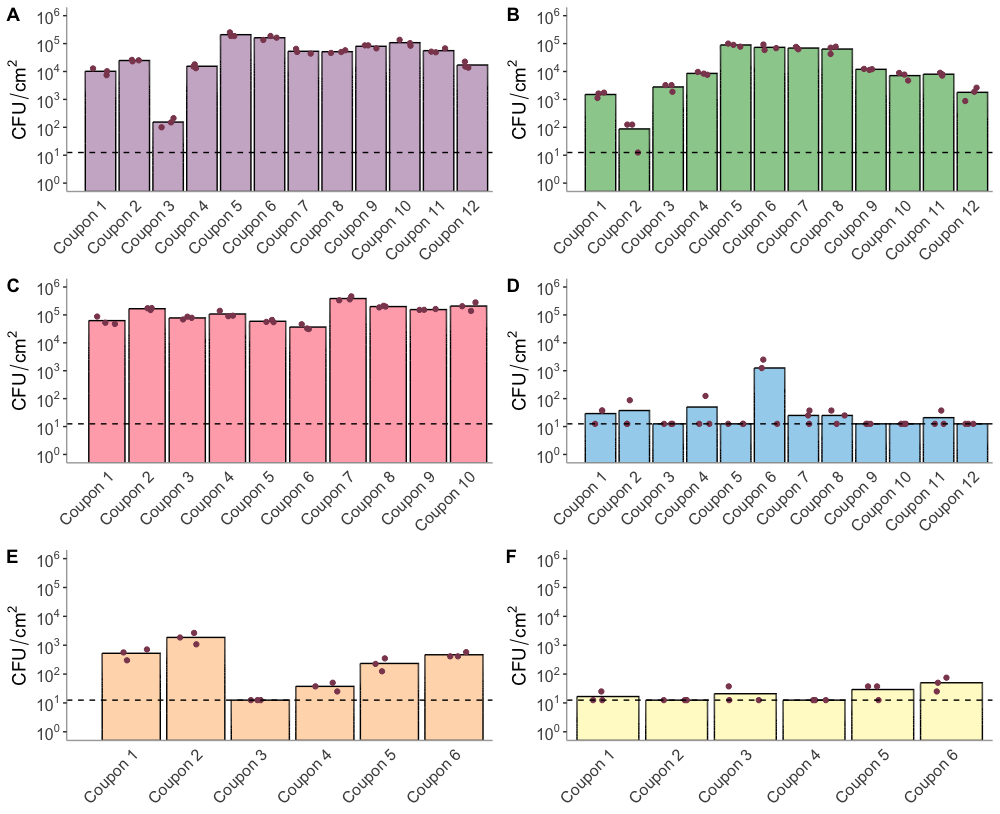


**Figure F1.** Transfer validation results. (A) Test ID 1 **–**  Baseline, (B) Test ID 2 **–**  90 minutes drying, (C) Test ID 3 **–**  1.5× concentrated TSA, (D) Test ID 4 **–**  5 minutes additional drying time post-touch, (E) Test ID 5 **–**  1-minute additional drying time post-touch, (F) Test ID 6 **–**  10^6^ cells/mL inoculum. Bars represent mean values. (•) represent individual values. Dashed line represents limit of detection (1.25 x 10^1^ CFU/cm^2^).


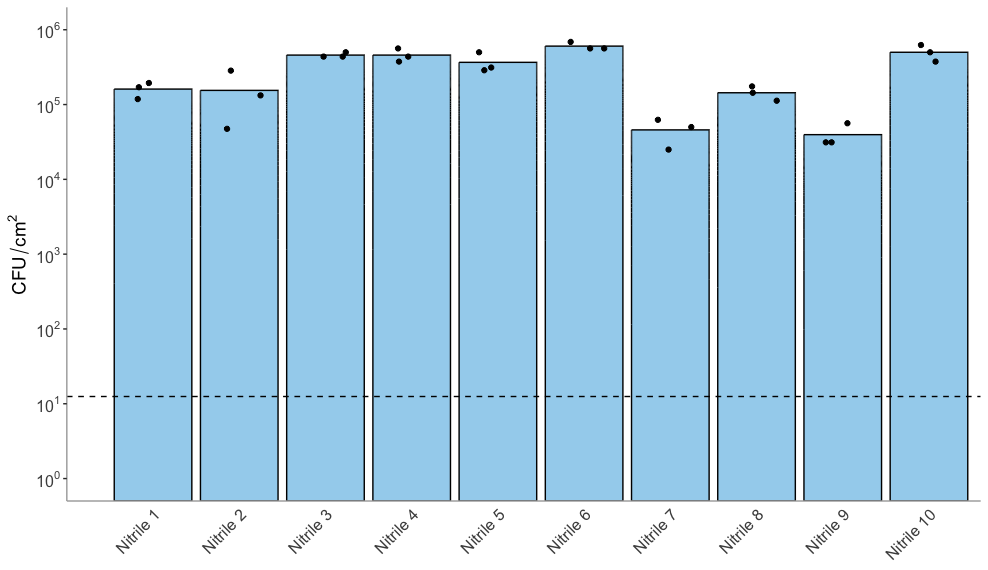


**Figure F2.** CFU recovery of MRSA on nitrile sections 5 minutes post-touch. Bars represent mean values. (•) represent individual values. Dashed line represents limit of detection (1.25 x 10^1^ CFU/cm^2^).
