# Supplementary figures and images for: A novel and validated 3D-printed method for the consistent and reproducible dry transfer of microorganisms for the determination of antimicrobial surface efficacy
Source: Appl Environ Microbiol. 2025 Jul 23;91(8):e00802-25. doi: 10.1128/aem.00802-25 (PMC12366365; doi:10.1128/aem.00802-25)

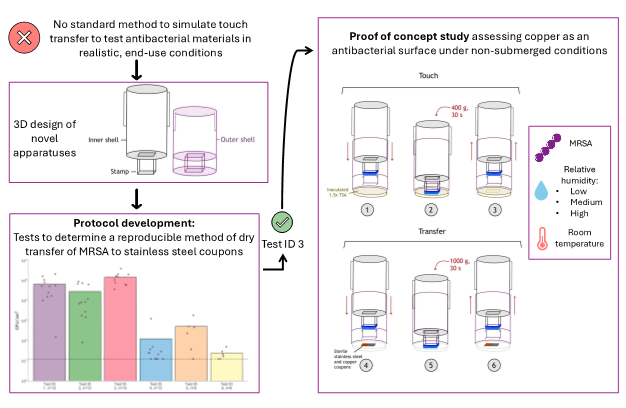

Supplement: Graphical abstract — Visual diagram of the study. [file aem.00802-25-s0007.tif]
